# Curcumin encapsulated zeolitic imidazolate frameworks as stimuli responsive drug delivery system and their interaction with biomimetic environment

**DOI:** 10.1038/s41598-017-12786-6

**Published:** 2017-10-03

**Authors:** Ashish Tiwari, Ashutosh Singh, Neha Garg, Jaspreet K. Randhawa

**Affiliations:** 1 School of Engineering, Indian Institute of Technology Mandi, Himachal Pradesh, India; 2 School of Basic Sciences, Indian Institute of Technology Mandi, Himachal Pradesh, India

## Abstract

Metal organic frameworks (MOFs) exhibit unique features of finely tunable pore structures, excellent chemical stability and flexible surface structural functionality, making them advantageous for a wide range of applications including energy storage, compound separation, catalysis, and drug delivery. The present work enlightens a novel approach of single step fabrication of CCM-ZIF-8 as a drug carrier and its application as stimuli responsive drug delivery systems via external stimuli involving change in pH and in presence of biomimetic cell membrane like environment using liposomes and SDS micelles. The methodology is devoid of any post synthesis drug loading steps. The synthesized curcumin encapsulated ZIF-8 frameworks demonstrate ultrahigh drug encapsulation efficiency (ca. 83.33%) and good chemical stability. *In vitro* drug release of curcumin was three times higher in acidic medium than in physiological pH. Cytotoxicity results demonstrated enhanced therapeutic effect of CCM-ZIF-8 than free curcumin. Confocal microscopy results confirmed the easy cellular internalization of CCM-ZIF-8 in HeLa cells. Intracellular distribution studies at various incubation times confirmed the clathrin-mediated endocytosis to lysosomal pathway of CCM-ZIF-8, but without mitochondria being an intracellular fate. The results signify that CCM-ZIF-8 is an efficient drug carrier for passive tumor therapy in future for cancer treatments.

## Introduction

Nanomedicine is expressively stepping forward in improving the efficiency of therapeutic drugs. The conventional approach of the drug delivery systems (DDS) was to encapsulate therapeutic drugs, protect them from degradation and control the drug release kinetics^1^. However, with the progress in understanding of human physiology and disease pathology, robust DDS are being developed that are capable of effective delivery of drugs at the site of disease while minimizing drug doses and reducing side effects^2^. Accordingly, a lot of research is now being focused towards achieving the overall control in drug bio-distribution, cell targeting, *in vivo* drug stability, controlling the drug release kinetics and understanding the drug release mechanism^3^. A number of drug carriers such as liposomes^4^, polymer nanoparticles^5^, micelles^6^, dendrimers^7^, and inorganic NPs^8^ specially iron oxide^9^, quantum dots^10^ and metal organic frameworks^11^ with diverse sizes, architectures and surface properties have been constructed and studied extensively. Nevertheless, all these carriers should have the preferred particle size of few hundreds of nanometers to allow systemic administration and ensure diffusion within the cell^12^.

Cancer is one of the world’s leading causes of death and its diversity and complexity involving variety of cell types, physiological distinctiveness, and extracellular matrices limits the therapeutics efficiency^13^. Cancer tissues are marked with more acidic environment with respect to body physiological tissues^14^. Although, various anti-cancer therapeutic agents are strategically designed, these molecules are either too large or highly charged, making the metabolically unstable, and/or too insoluble to target cancer cells^15^. Moreover, most of these chemotherapeutics are highly toxic and their systemic administration leads to serious side effects leading to significant destruction of normal cells along with cancerous cells. Hence, an efficient DDS must be designed to achieve effective cancer therapy via amalgamation of medicine with biology, chemistry, and engineering. The present study aims at developing smart DDS for anticancer drug curcumin.

Curcumin is a naturally occurring molecule having anticancer potential in addition to its anti-oxidative, anti-inflammatory, anti-HIV, and anti-angiogenic therapeutic properties^16^. These properties mainly arise due to its ability to form hydrogen bonds and undergo rotamerization^17^. However, due to its poor water solubility and fast degradation at physiological pH, there is a need for the development of an effective DDS capable of highly efficient drug delivery and selective cancer cell targeting^18^. This can be achieved by making use of advanced drug carriers that are able to control both the drug release and matrix degradation^19^. Metal organic frameworks (MOFs) have recently emerged as one of the most widely explored DDS^20^. MOFs are ordered porous solids which contain strong bonding between metals and organic linker molecules that endow robustness and form a well-defined geometrical framework structure^21^. MOFs can be synthesized by proper selection of metal cations and organic linkers for desirable physical and chemical functionalities^22^. In particular, nontoxic metals are confined with organic linkers to fabricate the MOFs structures for application as DDS. Biocompatible MOFs such as MILs, PCNs and ZIFs are extensively used as drug delivery vehicles^20,21^. However, very often the synthesis of MOFs involves high temperature, pressure and toxic solvents, limiting their application as DDS. Post-synthesis drug loading approaches are used to avoid drug degradation during synthesis under harsh conditions, leading to poor drug loading efficiencies. Several MILs and PCNs are not extensively studied in biomedical applications due to typical synthesis procedure, low drug encapsulation efficiency and large particle size prior to be used as drug carrier. This restrict the use of several MILs and PCNs for drug delivery applications.

Zeolitic imidazolate frameworks (ZIFs) are well-known for their biocompatible nature and remarkably large and available pores that can accommodate high amounts of drug^11^. Superior stability under aqueous physiological conditions, avoiding premature drug release and increased cellular uptake also benefit ZIF-8 as a drug carrier^23^. Moreover, its surface can easily be functionalized to tailor them for ‘active’ targeting to the diseased sites. The optical transparency of the ZIF-8 in visible and near-IR regions is an added advantage for an optically active drug such as curcumin for imaging purpose. In the present study, ZIF-8 synthesis and drug loading were achieved in a single step minimizing post drug loading processes. As a result, high drug encapsulation efficiency could be achieved.

Stimuli responsive drug delivery is an approach where numerous stimuli, including pH, temperature, enzymes, and reductive environment, can be used as site specific triggers for drug release^24^. As stated earlier, among all MOFs, zeolitic imidazolate frameworks (ZIF-8) have been popularly used as stimuli responsive drug carriers^25^. Sun *et al*. were the first one to demonstrate pH triggered release of 5-fluorouracil from ZIF-8 with nearly 70% release over 72 hrs^26^. Later, Liedana *et al*. synthesized caffeine encapsulated ZIF-8 frameworks and observed 28% of drug loading^27^. Zhuang *et al*. reported pH responsive drug release and anticancer activity using doxorubicin loaded ZIF-8 against MCF-7 cancer cell lines^28^. Zheng *et al*. investigated pH responsive drug release and anticancer activity using curcumin loaded ZIF-8 frameworks^29^. Adhikari and coworkers studied external stimuli responsive release of doxorubicin from ZIF-8 and ZIF-7 in biomimetic cell membrane like environment of sodium dodecyl sulfate micelles and liposomes^30^. Functionalized ZIF-8 frameworks have also been studied for pH responsive release of 5-fluorouracil by Ren *et al*.^23^.

The present work demonstrates a single step room temperature synthesis of curcumin encapsulated ZIF-8 (CCM-ZIF-8) frameworks. The method enables high drug loading (ca. 83.33%) in a short time of 15 min. Curcumin release from CCM-ZIF-8 using different external stimuli viz. pH of the medium and biomimetic cell membrane like environments was investigated thoroughly. The stimuli responsive drug release kinetics showed three-fold increase under low pH conditions as compared to human physiological conditions. Cytotoxicity experiments confirmed the enhanced therapeutic effects of CCM-ZIF-8 on HeLa cell lines. Intracellular colocalization of CCM-ZIF-8 upto 12 hrs confirmed the clathrin-mediated endocytosis to lysosomal pathway, without subsequent interaction with mitochondria. To the best of our knowledge, curcumin release kinetics in biomimetic cell membrane like environment has been studied for the first time. In addition, irregularities such as poor drug loading and burst drug release can be overcome by CCM-ZIF-8 as drug carrier in the present work.

## Results and Discussion

### Synthesis and Characterization of CCM-ZIF-8

**Figure 1:**
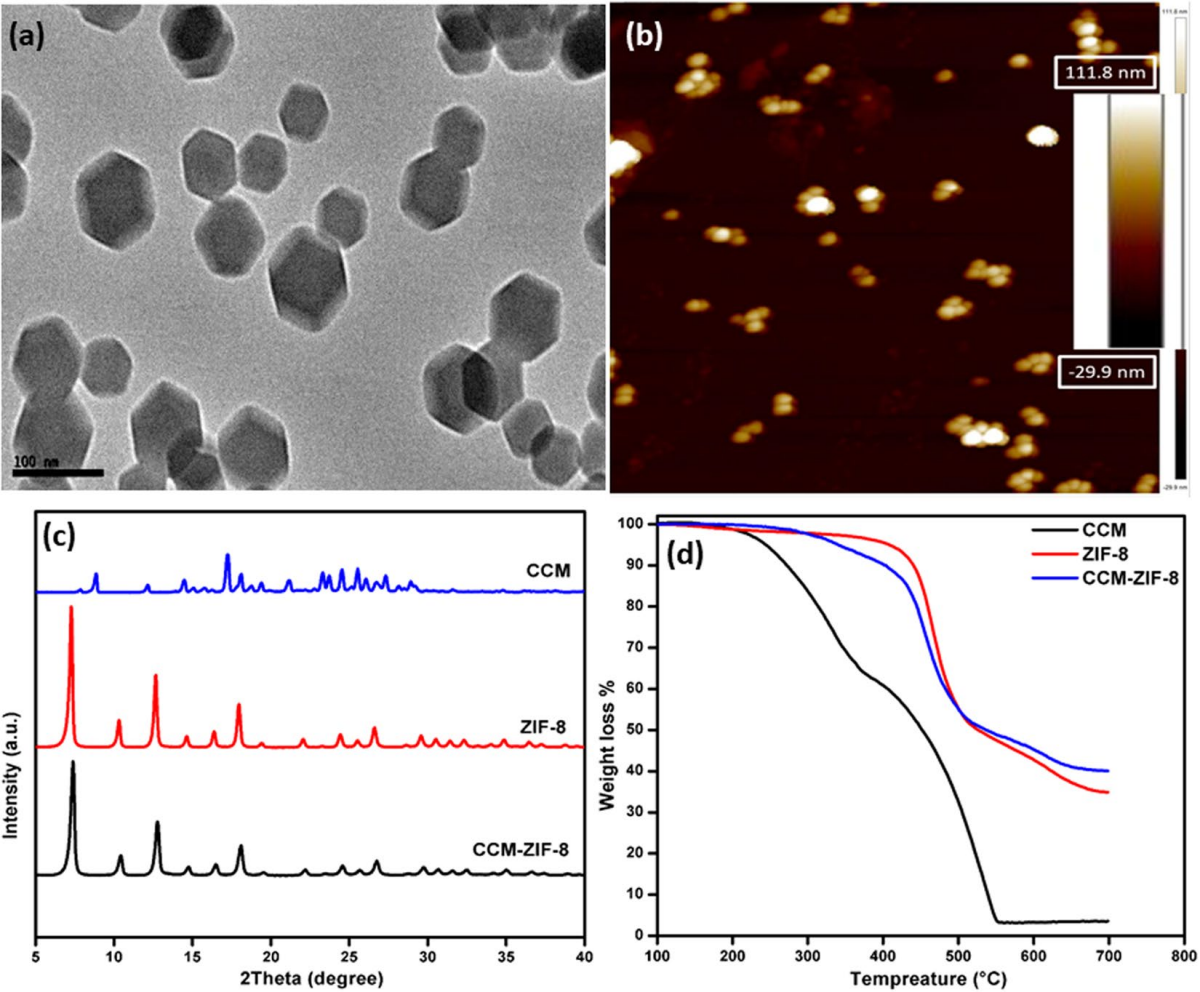
(**a**) TEM image of CCM-ZIF-8 (**b**) AFM image of CCM-ZIF-8 (**c**) PXRD spectra and (**d**) TGA thermograms of CCM, ZIF-8 and CCM-ZIF-8.

CCM-ZIF-8 were synthesized via a single step procedure as reported earlier^31^. The synthesis parameters were optimized as shown in Table S1 (supplementary information). Maximum encapsulation efficiency was achieved within 15 min of the reaction onset. Subsequent increase in reaction time did not show a significant increase in encapsulation efficiency indicating the occurrence of saturation beyond 15 min. Hence, we considered 15 min as optimum reaction duration for further studies. The hydrodynamic diameter of ZIF-8 and CCM-ZIF-8 was found to be 140 ± 5 and 145 ± 5 nm respectively and the corresponding particle size distribution is presented in Fig. S1 (supplementary information). Zeta potential of the ZIF-8 and CCM-ZIF-8 was +7.40 and +7.22 mV respectively. No significant change in surface charge of ZIF-8 on encapsulation of curcumin infers minimum surface adsorption of curcumin on ZIF-8. TEM images demonstrated the formation of rhombic dodecahedron particles having an average particle size of 80 ± 5 nm (Fig. 1a). AFM image in Fig. 1b and S9 (supplementary information) displayed smooth surface morphology of CCM-ZIF-8. The CCM-ZIF-8 showed no framework disintegration even upto 30 days indicating high stability of the synthesized frameworks as shown in Fig. S2 (supplementary information). X-ray diffraction patterns of pure curcumin, ZIF-8 and CCM-ZIF-8 are shown in Fig. 1c. The XRD pattern of ZIF-8 matched well with reported literature^32^.The characteristic peaks of ZIF-8 at 2θ values of 7.29, 10.44, 12.83 and 18.10 correspond to (110), (200), (211) and (222) index planes respectively. There was no change in the XRD pattern of CCM-ZIF-8 as compared to ZIF-8 indicating the framework stability and minimum impact on its crystallinity. Thermal characteristics of curcumin and ZIF-8 (Fig. 1d) matched well with the reported literature^33^. Curcumin starts to decompose at 200 °C and gets completely decomposed between 200 and ~550 °C whereas ZIF-8 is stable up to 500 °C. Encapsulation of curcumin in the ZIF-8 is clearly evident from the thermal curve of CCM-ZIF-8. An early mass loss ~300 °C can be attributed to the decomposition of curcumin that is encapsulated in ZIF-8 frameworks.

**Figure 2:**
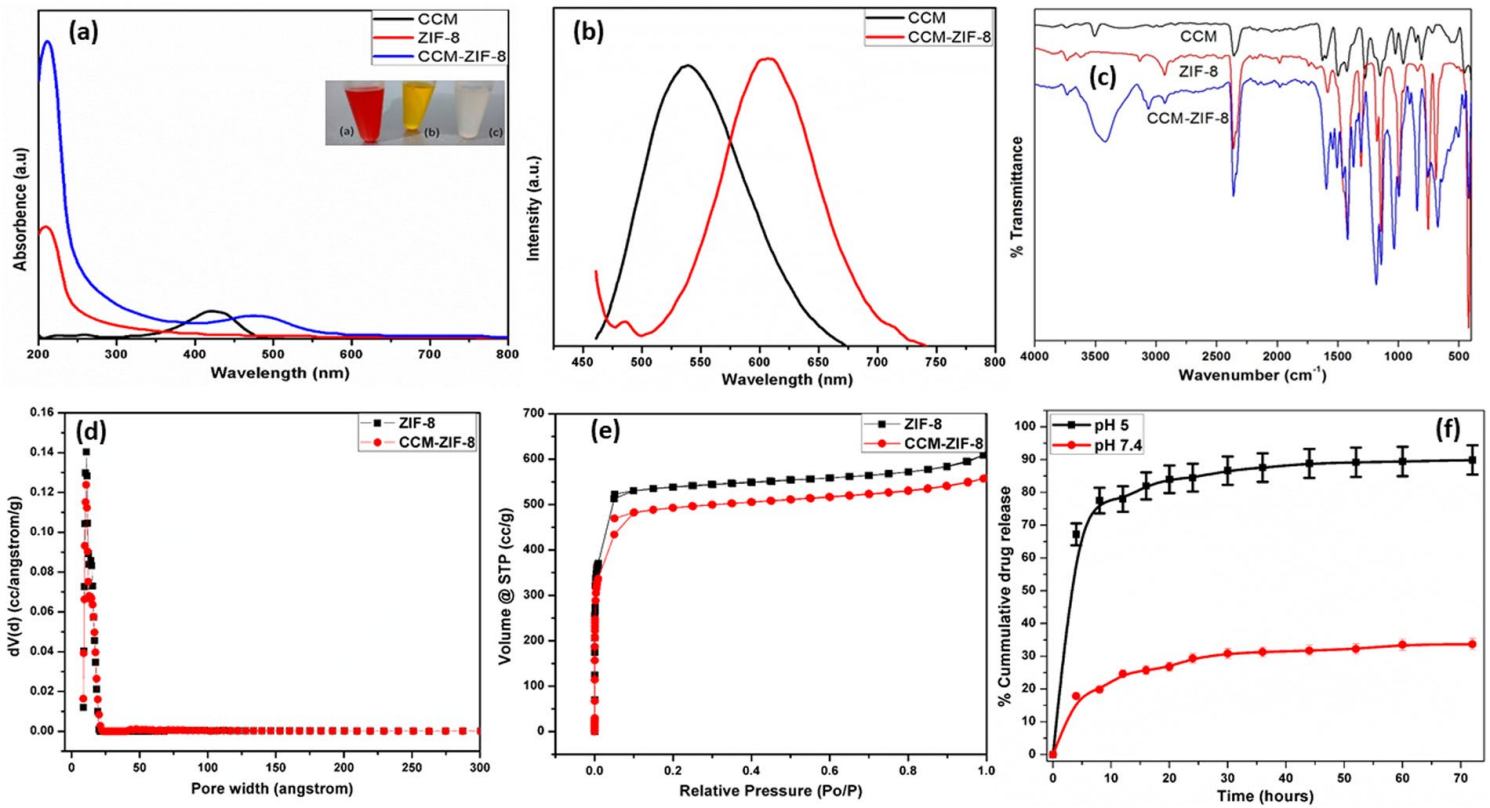
(**a**) UV-Vis absorption spectra of curcumin, ZIF-8 and CCM-ZIF-8. (Inset optical images of (**a**) CCM-ZIF-8, (**b**) Curcumin and (**c**) ZIF-8), (**b**) Photoluminescence spectra, (**c**) FTIR spectra, (**d**) Pore size distribution, (**e**) linear absorption desorption isotherm and (**f**) Percentage cumulative curcumin release in PBS solutions (0.5% Tween 20 at pH 5 and 7.4) at selected time intervals.

The encapsulation of curcumin into ZIF-8 frameworks was indicated by the change in yellow color of curcumin to orange (see Fig. 2a inset). UV-Vis and PL spectroscopic studies were also performed to confirm the curcumin encapsulation into ZIF-8. UV-Vis absorption spectra of pure ZIF-8, curcumin and CCM-ZIF-8 are shown in Fig. 2a. The characteristic absorption peaks of curcumin and ZIF-8 appeared at 425 and 213 nm respectively. The absorption peak at 425 nm in curcumin spectra is assigned to π-π* transitions which corresponds to enol form of curcumin. CCM-ZIF-8 spectra showed a red shift in curcumin peak to 485 nm which demonstrated the encapsulation of curcumin in ZIF-8 framework^34^. UV spectroscopy results correlated with the photoluminescence (PL) spectroscopy studies. The emission peak of pure curcumin was observed at 537 nm and it red shifts towards 610 nm in CCM-ZIF-8 as shown in Fig. 2b. FTIR spectra of pure curcumin, ZIF-8 and CCM-ZIF-8 are shown in Fig. 2c. The characteristic peaks of ZIF-8 were observed at 3135, 2928, 1606 and 1580 cm^−1^ corresponding to aromatic C-H stretching, aliphatic C-H stretching, C-C stretching and C-N stretching of imidazole respectively^35^. In curcumin spectra, the band at 3490 cm^−1^ corresponds to vibrations of free hydroxyl group of phenols. Other observed peaks at 1511, 1279 and 1152 cm^−1^ are attributed to C = C stretching of benzene ring, aromatic C-O stretching, and C-O-C stretching respectively^36^. In CCM-ZIF-8 spectrum, peaks at 3426, 3055, 2920, 2359, 1605, 1184, 1033 and 894 cm^−1^ correspond to OH stretching of phenol group, aromatic C-H stretching, aliphatic C-H stretching, C-C stretching, C-N stretching, C-O stretching, C-O-C stretching and C-C stretching respectively. However, stretching peak of phenolic group of curcumin was blue shifted from 3490 to 3426 cm^−1^ in CCM-ZIF-8 and confirm the encapsulation of curcumin in ZIF-8^34^. BET adsorption studies were performed to determine the surface area and porosity of ZIF-8 and CCM-ZIF-8. The BET isotherms as shown in Fig. 2d and e underline linear adsorption isotherm at low relative pressures. Significant increase in nitrogen uptake during adsorption experiments at low relative pressures confirmed microporosity of ZIF-8 and CCM-ZIF-8. Additionally, the decrease in BET surface area and pore volume of CCM-ZIF-8 (ca. 1922.6 m^2^g^−1^/0.762 cm^3^g^−1^) as compared to ZIF-8 (ca. 2229.3 m^2^g^−1^/0.827cm^3^g^−1^) also confirmed the effective encapsulation of curcumin in ZIF-8 frameworks. Raman spectroscopy as depicted in Fig. S3 (supplementary information) showed intense bands of ZIF-8 corresponding to methyl group and imidazole ring vibrations. The vibrational bands spotted at 168, 686, 1146 and 1458 cm^−1^correspond to Zn−N stretching, imidazole ring (C−N) stretching and methyl bending respectively^37^. Raman spectrum of pure curcumin confirmed the enol form due to the absence of (C = O) peaks in the1650–1800 cm^−1^ region. This observation matched well with ^1^H NMR spectral studies of curcumin (see Fig. S4, supplementary information). The aromatic (C = C) vibration bands appeared at 1601 and 1626 cm^−1^. In addition, bands at 1249, 1321 and 1430 cm^−1^ matched with enol (C-O), methyl (C-CH) and phenol (C-O) vibrations respectively^38^. However vibrational modes in ZIF-8 and CCM-ZIF-8 spectra almost remained identical which imply that there was no phase change in ZIF-8 after encapsulation of curcumin.

The ^1^H NMR spectra of ZIF-8 showed two peaks at δ 7.37 and δ 2.61 ppm corresponding to aromatic C-H protons and CH_3_ protons of imidazole respectively. Both these peaks were present in ^1^HNMR spectrum of CCM-ZIF-8 at δ 7.34 and δ 2.60 ppm respectively, albeit with slight chemical shift. ^1^H NMR spectra of pure curcumin displayed peaks at δ 5.95 and 5.93 ppm (in CD_3_OD and CD_3_OD + CF_3_COOD solutions respectively, Fig. S4, supplementary information) which correspond to methine protons (H_c_, scheme 1, supplementary information) and confirmed the enol form of curcumin. However, the same peak was absent in CCM-ZIF-8 spectra. It is due to the interaction between Zn and di-keto form of curcumin. This also affects the trans alkene protons (H_a_ and H_b_, scheme 1, supplementary information) adjacent to di-keto group which deshields and shows downfield in spectra with slight change in chemical shift. The detailed NMR spectra and respective chemical shift values of CCM and CCM-ZIF-8 are presented in Fig. S4 and Table S2 (supplementary information) respectively. ^1^H NMR spectra of curcumin and ZIF-8 were similarly reported in literature^39,40^. The presence of ^1^H NMR peaks of curcumin in CCM-ZIF-8 spectra successfully implied that curcumin was present in CCM-ZIF-8. From ^1^H NMR spectra, the relative intensities of methoxy peak in pure curcumin and CCM-ZIF-8 revealed that almost 3.2% of curcumin was present in the carrier system. This was in conformity with the drug loading capacity (3.42%) evaluated by UV-Vis spectra.

Curcumin changes its absorbance when bonded with metals and such change in absorbance can be used to detect curcumin in host molecules^16^. The interaction between curcumin and ZIF-8 was studied using fluorescence spectroscopy. Solutions of different concentrations of curcumin (1–10 µM) were added to the ZIF-8 and decrease in the emission peak at 310 nm was observed. Fluorescence quenching was calculated from Stern-Volmer equation (1)

1 $${I}_{o}/I=1+{K}_{sv}[M]$$

Where *I*_*o*_ and *I* are the fluorescence intensities of ZIF-8 before and after the addition of curcumin; *[M]* is the concentration of curcumin and *K*_*sv*_ is the quenching coefficient. Figure S5a and b (supplementary information) show a good linear relationship between ((*I*_*o*_ */I*) − *1*) and *[M]* in the range of 1–10 µM with a regression coefficient of 0.9745. The quenching constant *K*_*sv*_ is 1.77 × 10^−5^ M^−1^. The detection limit was calculated to be 1.2 µM based on a signal-to-noise ratio of 3^41^. The results highlight the use of ZIF-8 as a probe to detect curcumin at µM concentration.

The stimuli responsive drug release studies from CCM-ZIF-8 were performed under *in vitro* conditions at acidic and physiological pH mediums. It is established that cancer cells are acidic in nature^42^. Thus, drug release studies were performed at pH 5 and pH 7.4. The absorption peak of pure curcumin at 425 nm was used to make a calibration curve by serial dilution of known concentration of curcumin, as shown in Fig. S6 (supplementary information). The drug release studies were carried out for 72 hrs. Gradual increase in the absorbance at 425 nm with time was observed as shown in Fig. S7 (supplementary information). The cumulative drug release of 88% under acidic medium and 28% in physiological medium was achieved in 72 hrs (Fig. 2f). This shows three-fold increase in drug release under acidic medium as compared to physiological pH. This is due to protonation of imidazolate ions under acidic condition whereby, the co-ordination linkage between Zn and imidazolate ions breaks and leads to the effective increase in curcumin release.

pH stimuli responsive curcumin release from CCM-ZIF-8 was further probed under biomimetic cell membrane like environment of liposomes and SDS micelles^43^. The pK_a_ value of curcumin lies in between 7.9–10.5, and this indicates that curcumin is almost neutral at physiological conditions. SDS solutions of different concentrations, viz. above critical micelle concentration (CMC) (30 mM), at CMC (8 mM) and below CMC (3 mM) were prepared to observe curcumin release form CCM-ZIF-8 through fluorescence spectroscopy. The decrease in emission peaks was noticed with subsequent time. The percentage fluorescence quenching of emission peak intensity indicated the drug release and was calculated by using equation (2)

2 $$ \% Quenching=(Ii-If)/(Ii)\,\ast \,(100)$$

**Figure 3:**
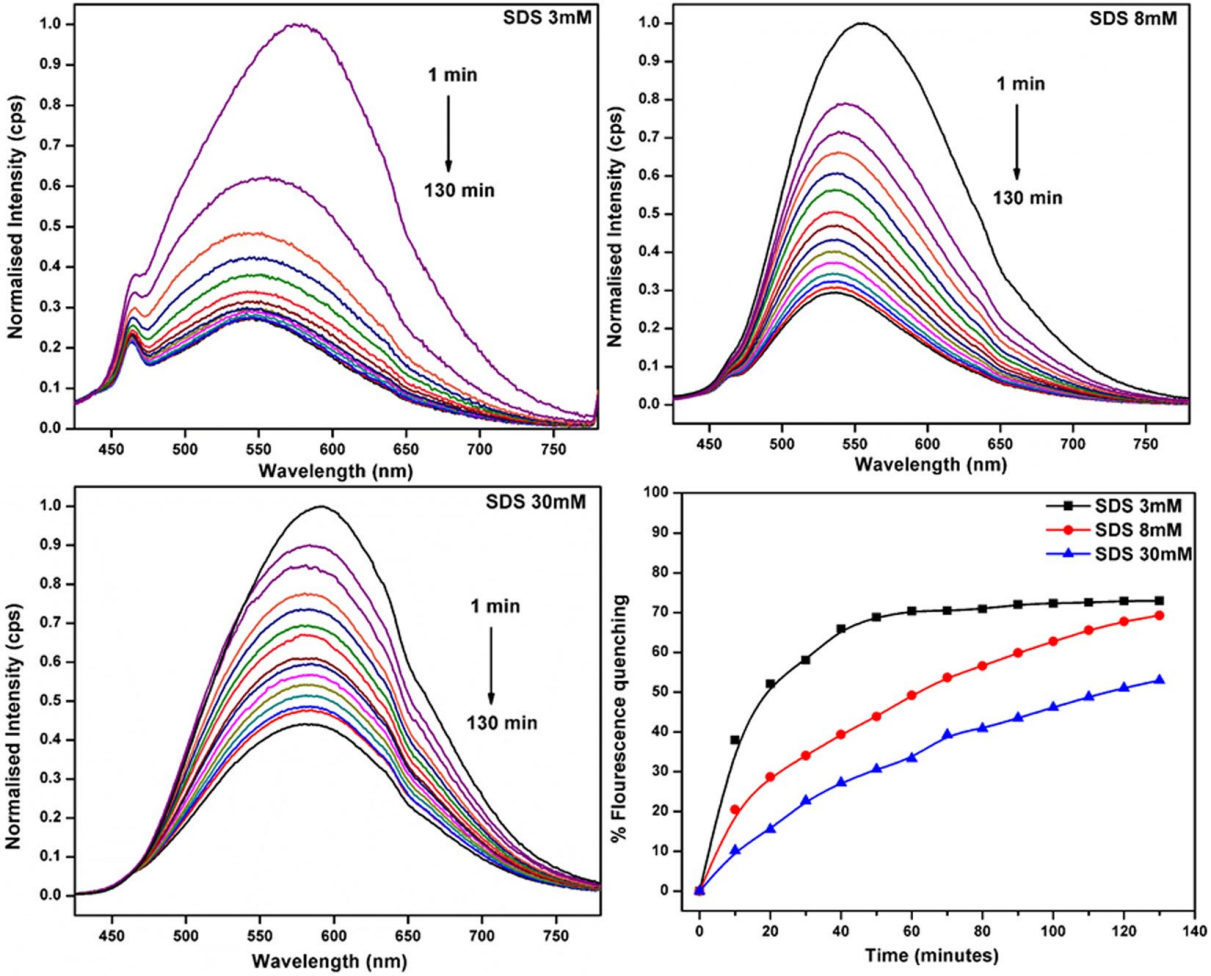
Fluorescence emission spectra of CCM-ZIF-8 in three SDS micelles solutions at concentration of 3, 8 and 30 mM with varying time from 1 to 130 minutes and comparison of ratio of percentage fluorescence quenching of emission peak.

**Figure 4:**
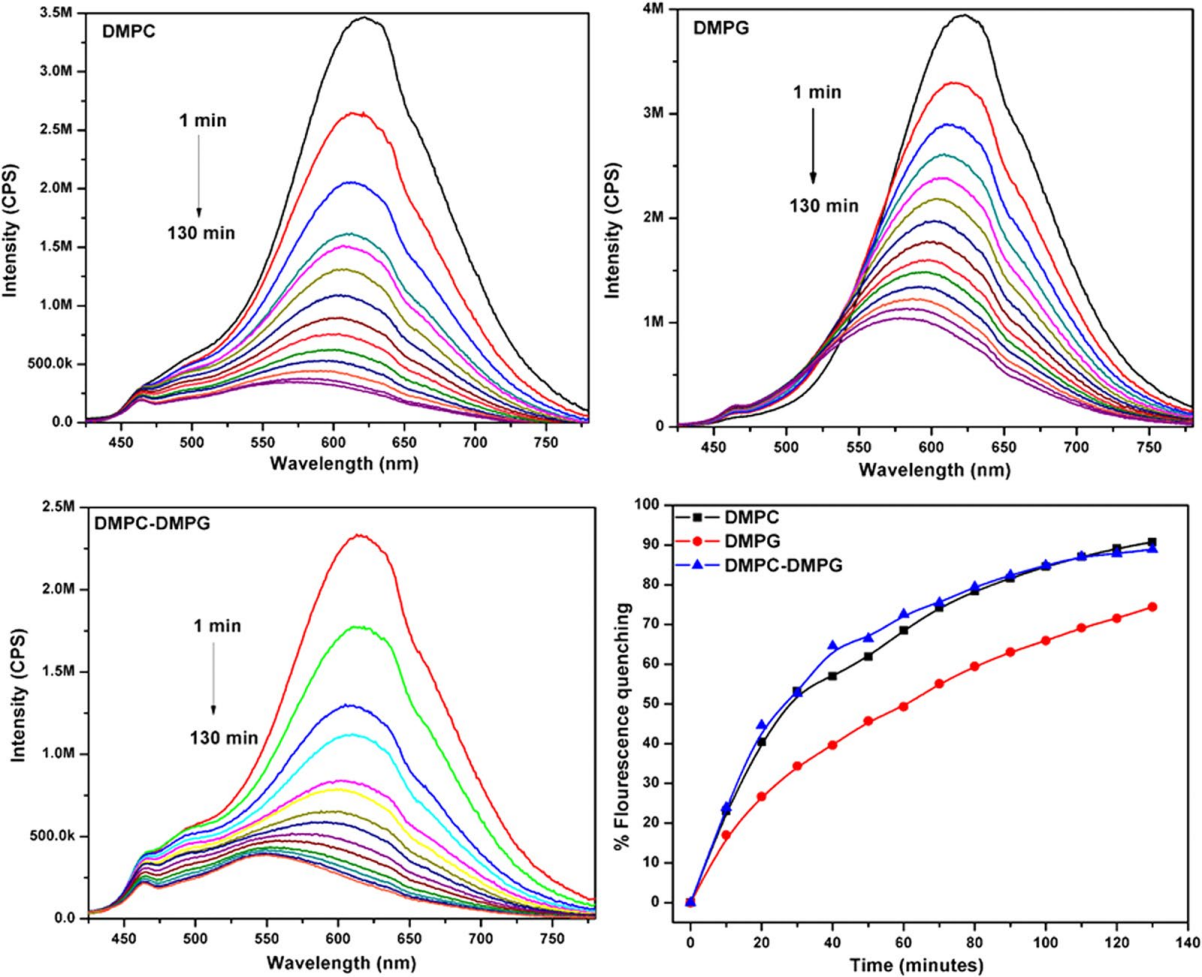
Fluorescence intensity emission spectra of CCM-ZIF-8 in three liposomes solutions of DMPC, DMPG, and DMPC: DMPG (9:1) at selected time intervals from 1 to 130 minutes and comparion of ratio of percentage fluorescence quenching of emission peak.

The percentage fluorescence quenching was observed for 3 mM (ca. 72.9%), 8 mM (ca. 70.9%) and 30 mM (ca. 60.6%) shown in Fig. 3. Additionally, emission peak of CCM-ZIF-8 at 580 nm showed blue shift with subsequent time in 3 and 8 mM SDS solutions but no such shift was observed in 30 mM. The fluorescence quenching behavior shows more hydrophobic interaction of CCM-ZIF-8 with SDS micelles above CMC (i.e. 30 mM). The bioadhesion of CCM-ZIF-8 with cell membrane like environment offers a strong stand towards its bioavailability. Thus, two commonly used lipids such as 1, 2-dimyristoyl-sn-glycero-3-phosphatidylcholine (DMPC) and 1, 2-dimyristoyl-sn-glycero-3-phospglycerol (DMPG) and both together DMPC: DMPG (9:1) were used for further studies. Figure 4 shows strong emission band of CCM-ZIF-8 in all the three liposomes. The emission peak shifts towards lower wavelength at 560, 580 and 550 nm in DMPC, DMPG and DMPC: DMPG (9:1) respectively from 625 nm. The maximum flouroscence quenching of CCM-ZIF-8 was observed in DMPC (ca. 90.76%) followed by DMPC:DMPG (9:1) (ca. 88.81%) and DMPG (ca. 74.4%). This specifies an electrostatic interaction between CCM-ZIF-8 and liposomes; as polarity of the medium influences the blue shift. The shift could be related to curcumin release and explains a strong synergy between CCM-ZIF-8 and liposomes. This clearly indicates that CCM-ZIF-8 is suitable for cellular internalisation under invitro cell uptake studies and could pave the way for therapeutic treatments.

### Cytotoxicity and cellular internalization studies

**Figure 5:**
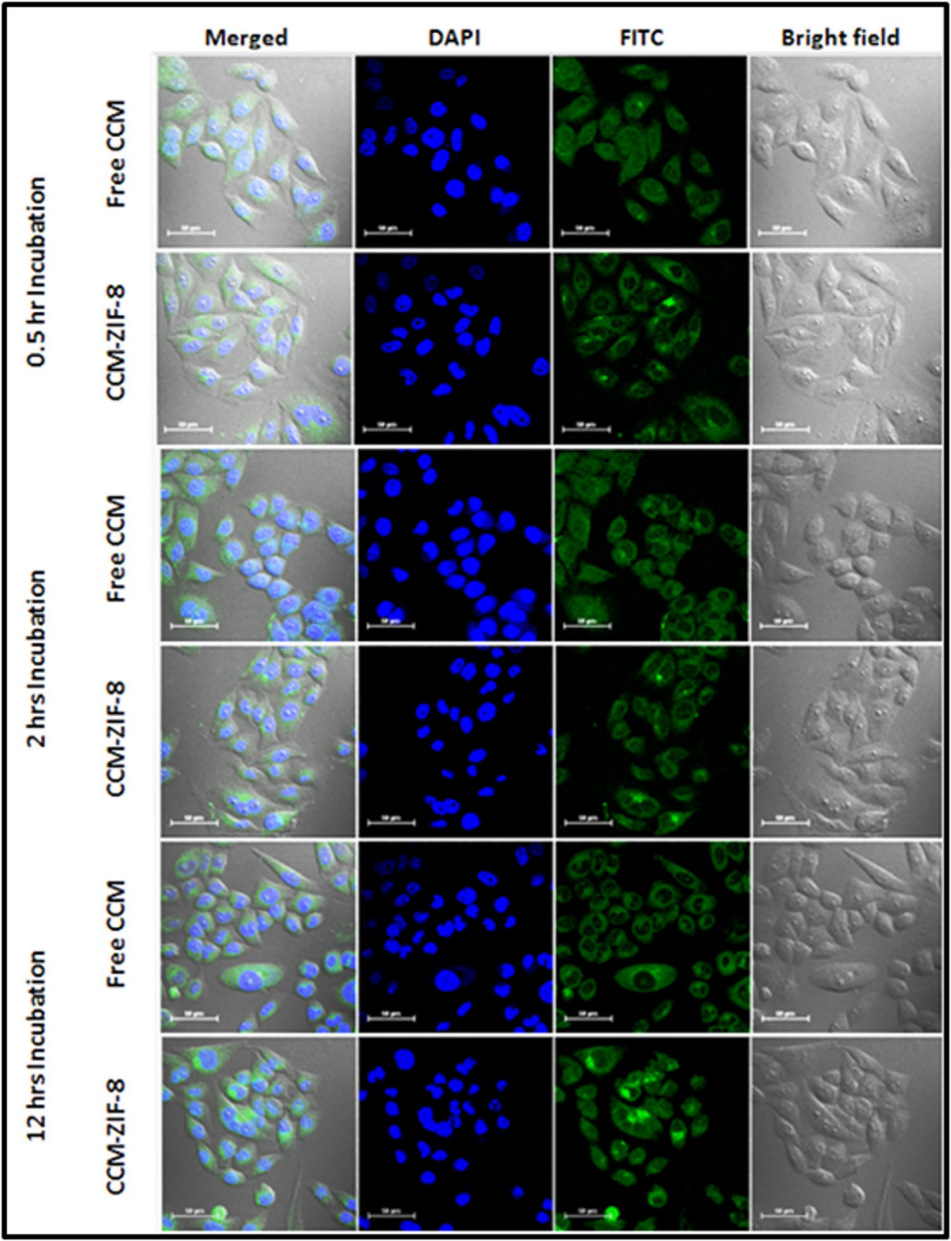
Confocal laser scanning microscopy (CLSM) images of HeLa cells incubated with free CCM and CCM-ZIF-8 at relative concentrations after 0.5, 2 and 12 hrs of incubation time. The right panel shows bright field fluorescence images, middle panel shows DAPI (blue fluorescence, stained by DAPI) and FITC channel (green fluorescence from CCM and CCM-ZIF-8) and left panel shows overlay merged fluorescence images of all channels on the same cells. (Scale Bar = 50 μm).

The observed photoluminescence spectra of CCM-ZIF-8 suggest that optical imaging and cellular internalization can be successfully attained through confocal laser scanning microscopy (CLSM) without any imaging probe. We evaluated cellular internalization of CCM-ZIF-8 in HeLa cell lines at three different incubation times viz. 0.5, 2 and 12 hrs. CLSM imaging in Fig. 5 clearly shows that green fluorescence in HeLa cells incubated with CCM-ZIF-8, implying cellular internalization. This highlights ZIF-8 as an efficient carrier protecting curcumin against degradation and enhancing the bioavailability by effective cellular internalization.

**Figure 6:**
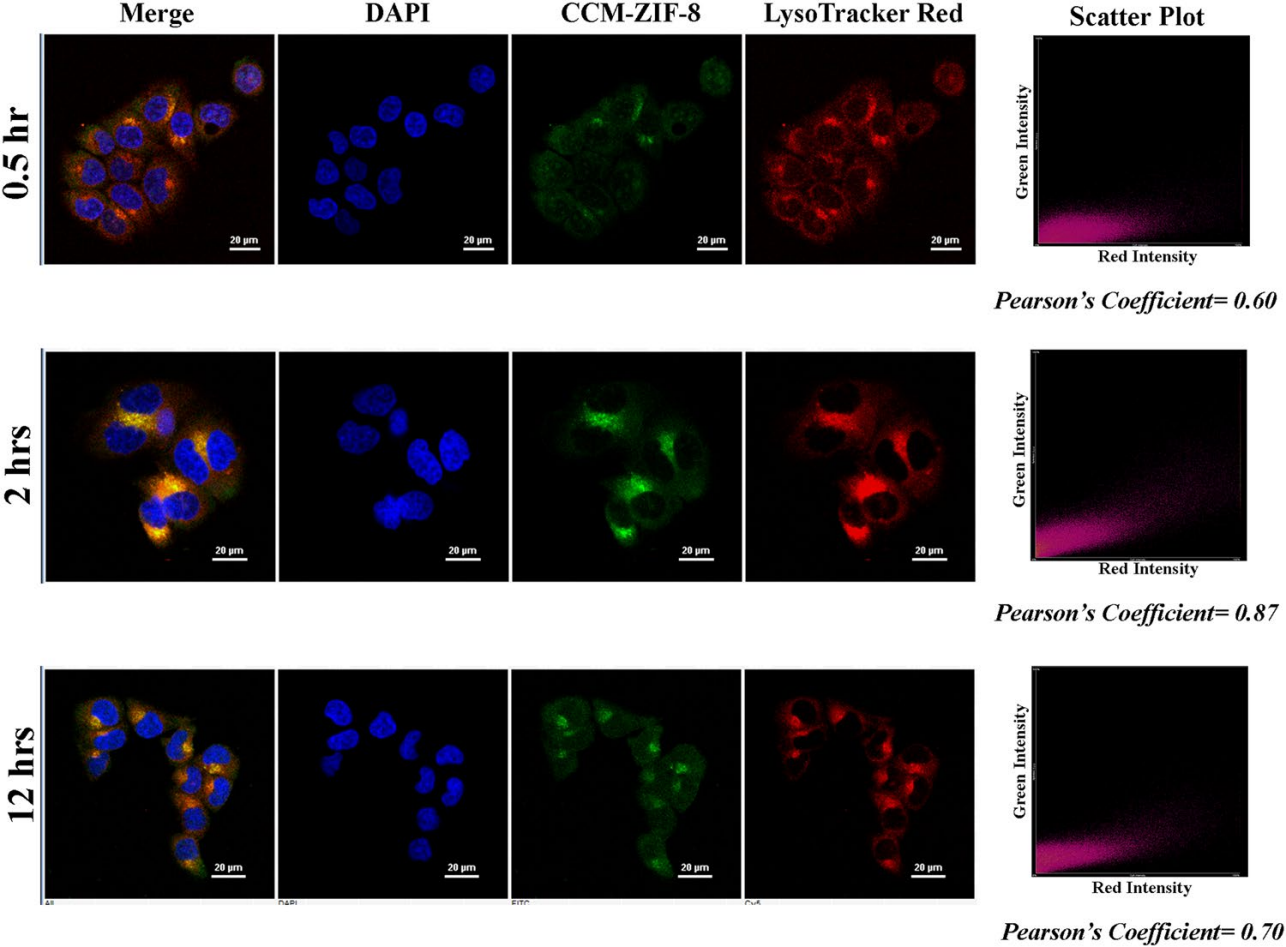
Co-localization of CCM-ZIF-8 and LysoTracker Red: HeLa cells were incubated for 0.5, 2 and 12 hrs with CCM-ZIF-8 (green), 0.5 h with Lysosome tracker (red) and the nuclei were stained by DAPI (for 15 min). CLSM images of HeLa cells with merged and individual images of DAPI, CCM-ZIF-8 and Lysosome tracker red, respectively (Scale bar = 20 µm). The scatter plot shows overlap between green and red channels and Pearson’s coefficient for the different time points are mentioned below the plot. Pearson coefficient is described with the maximum overlap between two channels, giving the value 1 and minimum overlap showing the value 0.

**Figure 7:**
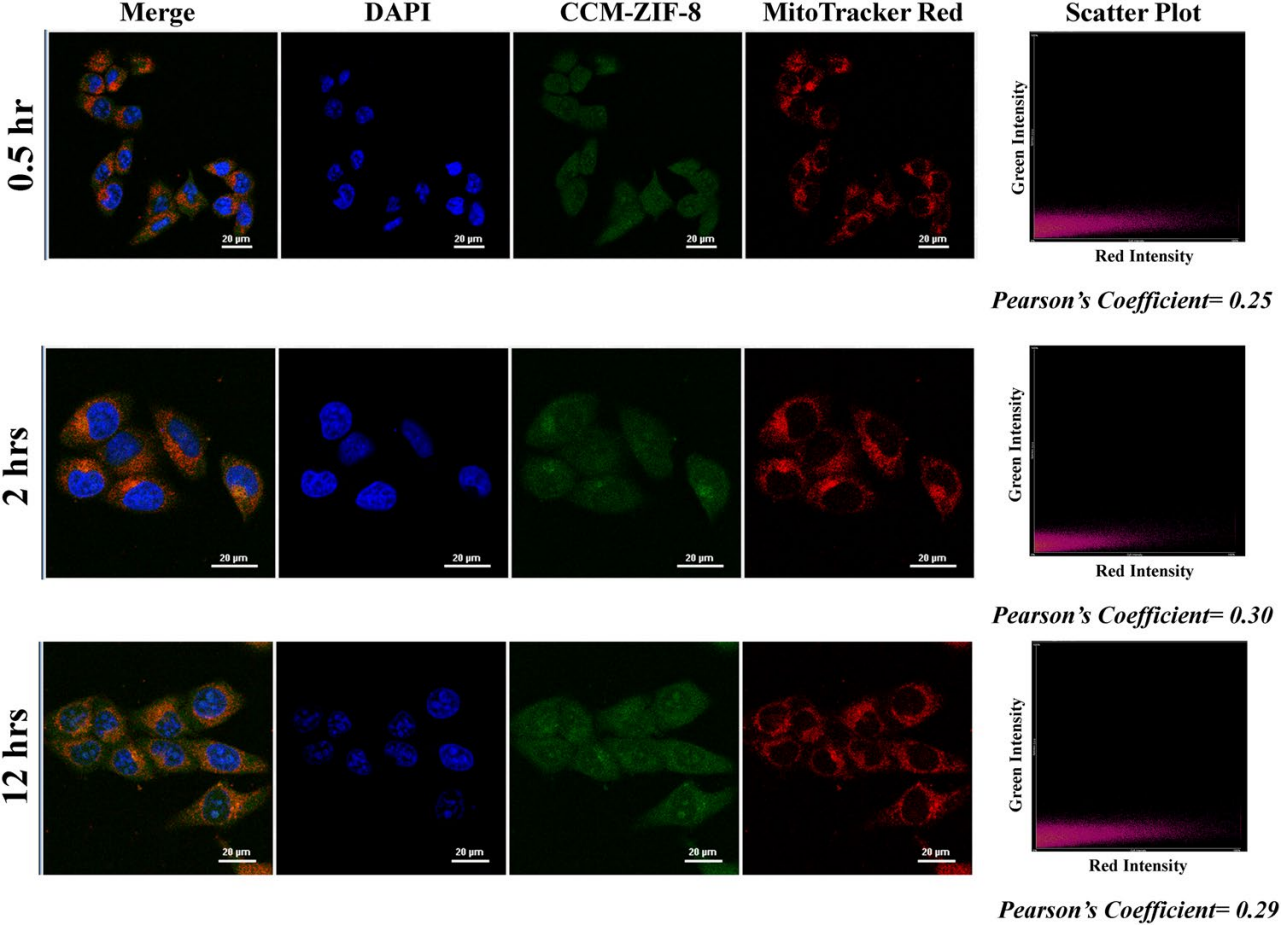
Co-localization of CCM-ZIF-8 and MitoTracker Red: HeLa cells were incubated for 0.5, 2 and 12 hrs with CCM-ZIF-8, MitoTracker Red (0.5 h), the nuclei were stained by DAPI (15 min). CLSM images shown are of HeLa cells with merged and individual images of DAPI, CCM-ZIF-8 and MitoTracker Red. (Scale bar = 20 µm). The Pearson Colocalization coefficient value is written below the scatter plot at individual time points.

Furthermore, we have examined the intracellular distribution of CCM-ZIF-8 at three different incubation times viz. 0.5, 2 and 12 hrs using two different cell organelle tracker dyes LysoTracker Red (for Lysosomes) and MitoTracker Red (for Mitochondria). Figure 6 shows the time dependent colocalization of LysoTracker Red and CCM-ZIF-8, wherein the green fluorescence of CCM-ZIF-8 is majorly overlapping with red fluorescence of LysoTracker Red after 2 hrs of incubation (Pearson’s coefficient = 0.87). With minimal Pearson’s coefficient values (Fig. 7), it is clear that CCM-ZIF-8 is not localized into mitochondria of HeLa cells, even after 12hrs of incubation. These results confirmed the clathrin-mediated endocytosis to lysosomal pathway of CCM-ZIF-8, with maximum colocalization with lysosome at 2 hrs, but without mitochondria being an intracellular fate. Our results are in accordance to the previous studies on the lysosomal fate of curcumin^44^.

**Figure 8:**
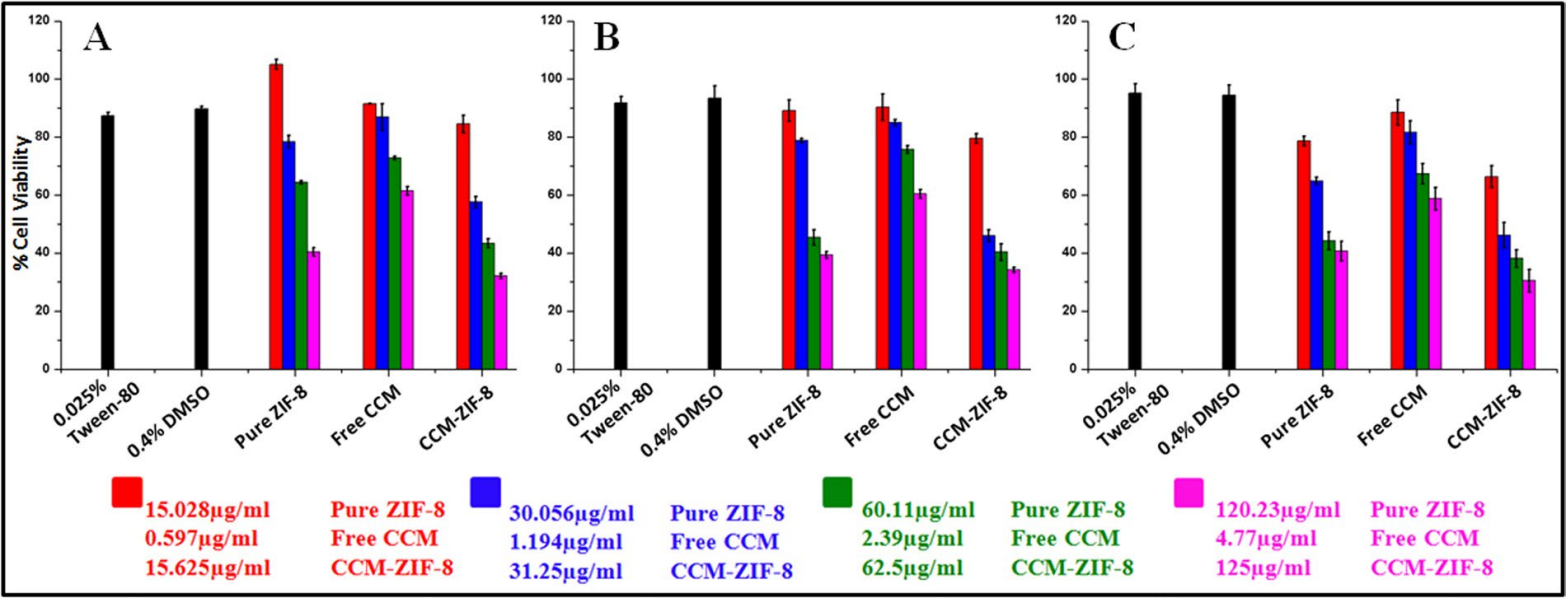
*In vitro* biocompatibility of ZIF-8 and cytotoxicity of free CCM and CCM-ZIF-8 against HeLa cell lines at various concentrations for 24 hrs (**A**), 48 hrs (**B**) and 72 hrs (**C**) of incubation time as assayed by MTT.

The cytotoxicity study using MTT assays was performed to evaluate the viability of free curcumin, ZIF-8 and CCM-ZIF-8 against HeLa cell lines. The cell viability and toxicity experiments were performed with 0.4% DMSO and 0.025% Tween 80 as positive controls. Prior to experiments, curcumin was dispersed in 0.4% DMSO whereas ZIF-8 and CCM-ZIF-8 were dispersed in PBS solutions containing Tween 80 (0.025%). The experiments were performed at different concentrations of curcumin, ZIF-8 and CCM-ZIF-8 in the range of 15.6 to 125 μg/mL for 24, 48 and 72 hrs (Fig. 8). It is clearly evident from the data shown in Fig. 8 that ZIF-8 has no obvious cytotoxicity upto 72 hrs when its concentration was below 50 μg/mL. However, a slight increase in cytotoxicity was observed at higher concentrations of pure ZIF-8 in all time points. On the other hand, CCM-ZIF-8 exhibited a strong cell growth inhibition effect on HeLa cells compared to free curcumin. High cytotoxicity of CCM-ZIF-8 than free curcumin was evident in all the concentrations at various time points. Only 62.5 μg/mL of CCM-ZIF-8 induced almost 57% of cell death compared to a mere 25% cell death caused by free curcumin (effective concentration = 2.39 μg/ml). The cytotoxicity was more pronounced at higher concentration of CCM-ZIF-8 as 70% of cell death was observed at 125 μg/mL. This must be due to the strong cellular internalization of CCM-ZIF-8 by HeLa cells. Additionally, the results also show a significant improvement in the bioavailability after the encapsulation of curcumin in ZIF-8. Moreover, using ZIF-8 as a drug carrier not only enhances cellular internalization but also ensures safe delivery of curcumin resulting in better cytotoxicity and bioavailability. This could be attributed to the small particle size and positive zeta potential of the present CCM-ZIF-8 frameworks. The small particle size favors the passive tumor targeting approach by enhanced permeability and retention (EPR) effect^45^. Additionally, positive zeta potential could lead to enhanced electrostatic interaction with the cell membranes, thereby improving cellular internalization of drug carriers.

## Conclusion

We demonstrated successful encapsulation of a hydrophobic drug curcumin inside ZIF-8 frameworks via a single step synthesis process avoiding any kind of post synthesis drug loading. The small size of CCM-ZIF-8 was found to be optimal for cellular uptake through EPR effect in cancer cells. A high drug encapsulation efficiency of 83.3% was achieved. The characteristic pH stimuli responsive behavior of ZIF-8 can be used to trigger drug release in external stimuli change in pH and biomimetic cell membrane like environment of micelles and liposomes. *In vitro* drug release results showed almost 88% drug release over a period of 72 hrs in acidic conditions. Cytotoxicity and cellular internalization results displayed improved therapeutic effect of CCM-ZIF-8 on HeLa cancer cell lines. CCM-ZIF-8 showed intracellular internalization via clathrin-mediated endocytosis to lysosomal pathway. With this work, we conclude that CCM-ZIF-8 improves the bioavailability of curcumin and enhance its therapeutic effect via efficient cellular internalization and show pH responsive drug release.

## Experimental

### Materials and Methods

Curcumin, zinc nitrate hexahydrate, 2 methyl imidazole, Tween 20 and 80, Phosphate buffer saline (PBS), DMPG, DMPC and Sodium dodecyl sulphate (SDS) were purchased from Sigma Aldrich. All chemicals were used without any further purification. The water used in all experiments was of Millipore Milli-Q grade.

### Synthesis of curcumin encapsulated ZIF-8 (CCM-ZIF-8)

Initially, 150 mg of zinc nitrate hexahydrate was dissolved in 5 ml deionized water. In another beaker, 330 mg of 2-methyl imidazole and 5 mg of curcumin were dissolved in 10 ml of methanol with continuous stirring till complete dissolution occurs. The two solutions were then mixed and stirred for various time intervals. A color change from colorless to orange was observed immediately indicating the formation of CCM-ZIF-8 drug carriers. The solution was centrifuged at 13000 rpm for 30 min and washed 3 times with methanol. The synthesized CCM-ZIF-8 was suspended in methanol for further characterization. The pure ZIF-8 drug carriers were also synthesized by similar synthesis procedure.

### Estimation of drug loading

Appropriate amount of dried CCM-ZIF-8 sample was decomposed in 50 µL of hydrochloric acid (2 M) and diluted to 2 ml with ethanol. Then, the solution was examined by UV-Vis spectroscopy at wavelength of 425 nm with the standard calibration curve as shown in Fig. S6 (supplementary information). The drug loading capacity and drug loading efficiency were calculated using following equations:

$${\rm{DLE}}( \% )=({\rm{amount}}\,{\rm{of}}\,{\rm{loaded}}\,{\rm{drug}})/({\rm{total}}\,{\rm{amount}}\,{\rm{of}}\,{\rm{feeding}}\,{\rm{drug}})\times 100 \% $$

$${\rm{DLC}}( \% )=({\rm{amount}}\,{\rm{of}}\,{\rm{loaded}}\,{\rm{drug}})/({\rm{amount}}\,{\rm{of}}\,{\rm{drug}}\,{\rm{loaded}}\,{\rm{NPs}})\times 100 \% $$

### Characterization of CCM-ZIF-8

The crystalline phase of the prepared frameworks was analyzed using powder x-ray diffraction (Rigaku smart lab) with CuKα (λ = 1.5418 Å) radiation. The samples were scanned between 5–40° 2θ range. The morphology of the samples was characterized by transmission electron microscopy (TEM) using FEI Tecnai TEM equipped with a LaB_6_ source operating at 200 kV. The AFM imaging was done using Dimension ICON (Bruker) AFM in tapping mode at scanning rate of 0.9 Hz. UV-Vis absorption spectra were measured with Shimadzu U-2450 UV-Vis spectrophotometer in the wavelength range of 200–800 nm. Fluorescence spectra were recorded by Cary fluorescence spectrophotometer (Agilent Technologies) in the wavelength range of 200 to 800 nm. FTIR spectra were recorded with Agilent Technologies Cary 6000 series FTIR spectrometer at wavenumber from 400 to 4000 cm^−1^. The particle size distribution and zeta potential was measured by dynamic light scattering (Malvern Zetasizer). The Brunuer-Emmett-Teller (BET) surface area measurements were performed on Quanta chrome, automatic volumetric instrument with pressure ranging from 0 to 760 Torr with low-pressure volumetric nitrogen adsorption measurements. Thermal properties were measured by Perkin Elmer Pyris Thermogravimetric (TGA) analyzer under nitrogen atmosphere from room temperature to 700 °C with the heating rate of 5 °Cmin^−1^. Raman studies were performed using a Horiba LABRAM high resolution UV-Vis-NIR Raman spectrophotometer equipped with a confocal microscope. The samples were analyzed using a 534 nm He-Ne laser employing a 10X objective lens to achieve an average spot size of 3 µm diameter. ^1^H NMR spectra were collected on a Bruker Advance III 500 MHz NMR spectrometer in CD_3_OD and CD_3_OD and CF_3_COOD solvents. The curcumin content was calculated by using the following formula,

$$\begin{array}{c} \% {\rm{CCM}}=({\rm{intensity}}\,{\rm{of}}\,{\rm{methoxy}}\,{\rm{peak}}\,{\rm{in}}\,\mathrm{CCM} \mbox{-} \mathrm{ZIF} \mbox{-} 8)/({\rm{intensity}}\,{\rm{of}}\,{\rm{methoxy}}\\ \,{\rm{peak}}\,{\rm{in}}\,{\rm{pure}}\,{\rm{CCM}}+{\rm{intensity}}\,{\rm{of}}\,{\rm{methoxy}}\,{\rm{peak}}\,{\rm{in}}\,\mathrm{CCM} \mbox{-} \mathrm{ZIF} \mbox{-} 8)\times 100 \% \end{array}$$

### *In vitro* drug release studies

Drug release with pH stimuli under acidic and physiological conditions were carried out in a solution of phosphate buffer saline (PBS) and Tween 20 (0.5 v/v %)^46^. For the drug release studies, initially 2 mg of CCM-ZIF-8 was dissolved in 20 ml of PBS and Tween 20 solution (pH 5 and 7.4) under continuous shaking (120 rpm) at 37 °C. After selected time intervals, 1 mL of solution was taken by centrifuging and replaced with fresh PBS solution. The amount of released curcumin was measured by UV-Vis spectroscopy at wavelength of 425 nm with the calibration curve. Percentage cumulative release was also calculated at selected time intervals.

### Preparation of Liposomes

The liposomes and SDS micelles were prepared using a method reported elsewhere^43^. The aqueous PBS solution was taken in round bottom flask, and heated above the phase transition temperature of the used lipids. On the other hand, the required amount of lipids was dissolved in the ethanol solution (less than 1% v/v) and stirred till lipids were completely dissolved. Subsequently, the lipid ethanol solution was immediately injected into the prepared PBS solution above the phase transition temperature of lipids under continuous stirring.

### Cell culture preparation

HeLa cell lines were procured from National Centre for Cell Sciences (NCCS), Pune, India and were cultured in DMEM (GibcoTM) supplemented with 10% of fetal bovine serum (FBS) and 1% penicillin-streptomycin (GibcoTM) in a humidified CO_2_ (5%) incubator at 37 °C temperature and passaged using Trypsin (GibcoTM) when the cells become confluent.

### Cytotoxicity Assay

A total of 150 µl of cells suspended in 1% FBS were seeded in 96 well plate at a density of 3000 cells/well and allowed to adhere for 24 hrs. The concentration of free CCM and pure ZIF-8 that were used was in relative to the loading percentage of CCM in CCM-ZIF-8. DMEM with 0.025% Tween-80 (for pure ZIF-8 and CCM-ZIF-8) and 0.4% Dimethyl sulfoxide (DMSO) (for free CCM) were used to disperse the samples. Treated cells were incubated for 24, 48 and 72 hrs at 37 °C. 0.025% Tween-80 and 0.4% DMSO were used as vehicle control along with positive (only cells) and negative (only media) controls. 20 µl of 3- (4, 5-dimethylthiazol-2-yl) -2, 5- diphenyl tetrazolium bromide (MTT) was added to each well and further incubated for 3hrs followed by the addition of 100 µl of DMSO to each well. Absorbance was read at 570 nm with 650 nm as reference.

### Cellular uptake study

300 µl of HeLa cell suspension was seeded in a 6 well plate on a sterile cover slip (22 × 22 mm) and allowed to adhere for 24 hrs. The cells were then incubated with 15.625 µg/ml of CCM-ZIF-8 and 0.597 µg/ml of CCM (equivalent to the loading percentage of CCM in 15.625 µg/ml of CCM-ZIF-8) for 0.5, 2 and 12 hrs. After each incubation, the medium was removed, cells were washed 3 times with PBS, and fixed with 4% paraformaldehyde. Cells were further counterstained with DAPI. The cells were mounted on microscopic slides and imaged under Nikon Eclipse Ti-U inverted microscope.

### Intracellular localization study

To study the intracellular localization of CCM-ZIF-8, the adhered HeLa cells were treated with 20 µg/mL of CCM-ZIF-8 for 0.5, 2 and 12 hrs. The LysoTracker Red/ MitoTracker Red (obtained from ThemoFisher) was added to the cells containing CCM-ZIF-8 and incubated for 30 min. Afterwards, the cells were washed with PBS, fixed with 4% paraformaldehyde and counter stained with DAPI. The cover slips were mounted over microscopic slides and studied under Nikon Eclipse Ti-U inverted microscope. The colocalization analysis tool of NIS-Element (Nikon microscope software) was used to calculate the Pearson colocalization coefficient of CCM-ZIF-8 in comparison to LysoTracker Red/ MitoTracker Red.

### Data Availability Statement

All the data generated or analyzed during the study are included in this published article (and its supplementary information files).

## Electronic supplementary material

Curcumin encapsulated zeolitic imidazolate frameworks as stimuli responsive drug delivery system and their interaction with biomimetic environment
