# Supplementary material for: Curcumin encapsulated zeolitic imidazolate frameworks as stimuli responsive drug delivery system and their interaction with biomimetic environment
Source: Sci Rep. 2017 Oct 3;7:12598. doi: 10.1038/s41598-017-12786-6 (PMC5626696; doi:10.1038/s41598-017-12786-6)
Supplement: Supplementary file 1 — Curcumin encapsulated zeolitic imidazolate frameworks as stimuli responsive drug delivery system and their interaction with biomimetic environment [file 41598_2017_12786_MOESM1_ESM.doc]

Electronic supporting information

**Curcumin encapsulated zeolitic imidazolate frameworks as stimuli responsive drug delivery system and their interaction with biomimetic environment**

Ashish Tiwari^a^,Ashutosh singh^b^, Neha Garg*, and Jaspreet K Randhawa*^a^

^a^School of Engineering, Indian Institute of Technology Mandi, Himachal Pradesh, India

^b^School of Basic Sciences, Indian Institute of Technology Mandi, Himachal Pradesh, India

*Corresponding author postal address

Dr. Jaspreet Kaur Randhawa, School of Engineering,

Indian Institute of Technology Mandi,Mandi-175005,India

Telephone: +91-1905-267056

Email: jaspreet@iitmandi.ac.in

**Table S1.**Synthesis parameters.

**Figure S1.**DLS size distribution of ZIF-8 and CCM-ZIF-8.

**Figure S2**.TEM images of CCM-ZIF-8 after suspending 30 days in methanol solution.

**Figure S3**.Raman spectra of curcumin, ZIF-8 and CCM-ZIF-8.

**Scheme 1.**Schematic representation of curcumin before and after encapsulated inside ZIF-8.

**Table S2**. Numerical values calculated in ^1^H NMR spectra of curcumin and CCM-ZIF-8.

**Figure S4.**^1^H NMR spectra of CCM (in pure CD_3_OD), CCM (in CD_3_OD and CF_3_COOD), ZIF-8 (in CD_3_OD and CF3COOD) and CCM-ZIF-8 (in CD_3_OD and CF_3_COOD).

**Figure S5.**Fluorescence spectra of ZIF-8 at various concentrations of curcumin ranging from 1-10 µM and linear relationship curve.

**Figure S6.**UV-Vis absorption spectra of curcumin in ethanol at different concentrations. The Linear relationship for concentration and absorbance in ethanol, range of concentration is from 0.04-20 µg/ml.

**Figure S7.**The absorption peaks of curcumin recorded at 425 nm in PBS solutions (pH 5 and 7.4) at selected time intervals in UV-Vis spectra during drug release studies.

**Figure S8.**The optical images of curcumin and CCM-ZIF-8 under UV light excitation at 380 nm.

**Figure S9.** AFM images of CCM-ZIF-8.

**Experimental Section**

Synthesis of CCM-ZIF-8 follows reported method with some modifications [1]. The aqueous solution of Zn(NO_3_)_2_ (150mg/5mL) was added to 2-MIM (330 mg) and CCM (5 mg) in methanol solution (10mL). CCM-ZIF-8 were obtained after stirring upto 30 min. With the highest drug loading sample (DLC and DLE to be 3.42% and 83.33%), particle size was foundin an average of 80 nmfrom TEM images.

Table S1: The reaction time and calculated DLE and DLC of different samples of CCM-ZIF-8. The concentration of curcumin, zinc nitrate and 2 methyl imidazole remained same in all the synthesis.

|  | Reaction time  (min) | % DLE | % DLC |
| --- | --- | --- | --- |
| Sample 1 | 1 | 34.0 | 1.26 |
| Sample 2 | 2 | 36.62 | 1.98 |
| Sample 3 | 5 | 43.32 | 2.56 |
| Sample 4 | 10 | 66.68 | 3.16 |
| Sample 5 | 15 | 83.33 | 3.42 |
| Sample 6 | 20 | 79.34 | 3.04 |
| Sample 7 | 30 | 82.72 | 2.95 |


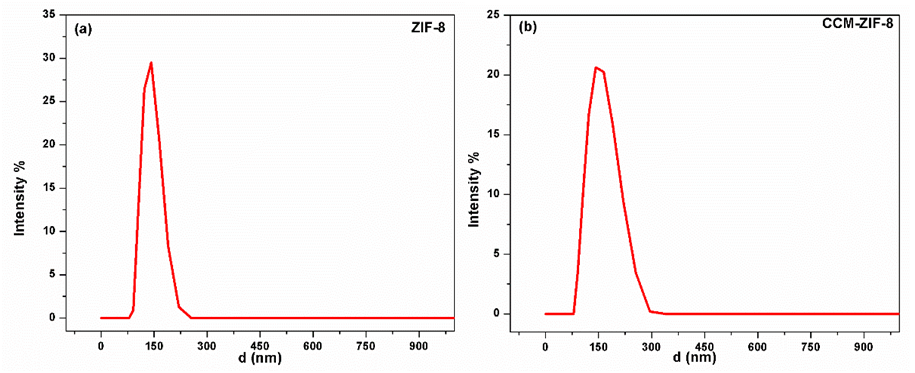


Figure S1: Particle size distribution of ZIF-8 (a) and CCM-ZIF-8 (b).


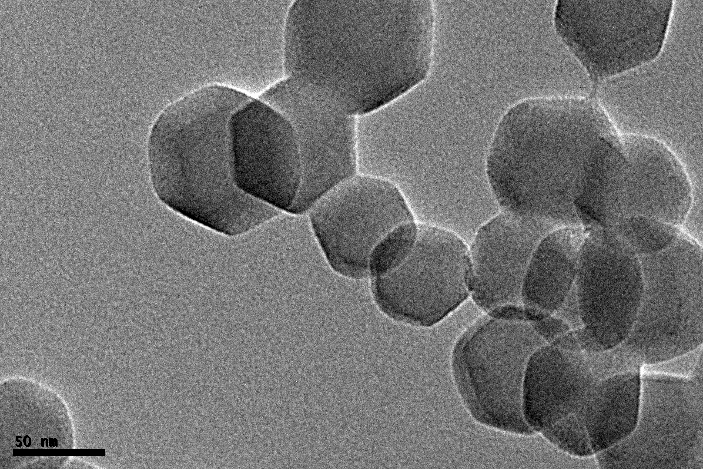


Figure S2: TEM image obtained from suspended solution of CCM-ZIF-8 in methanol after 30 days.


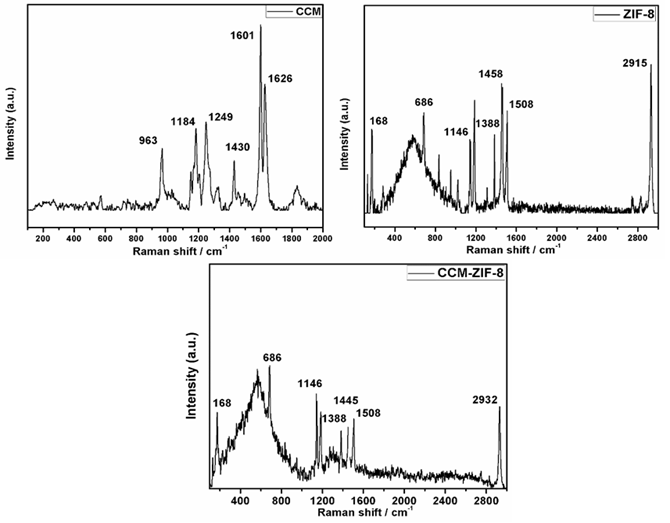


Figure S3: Raman spectraof curcumin, ZIF-8 and CCM-ZIF-8.

**^1^H NMR spectroscopy**

^1^H NMR spectroscopic analysis of CCM and CCM-ZIF-8 successfully displayed enol to diketotransformation of curcumin during the formation of CCM-ZIF-8.


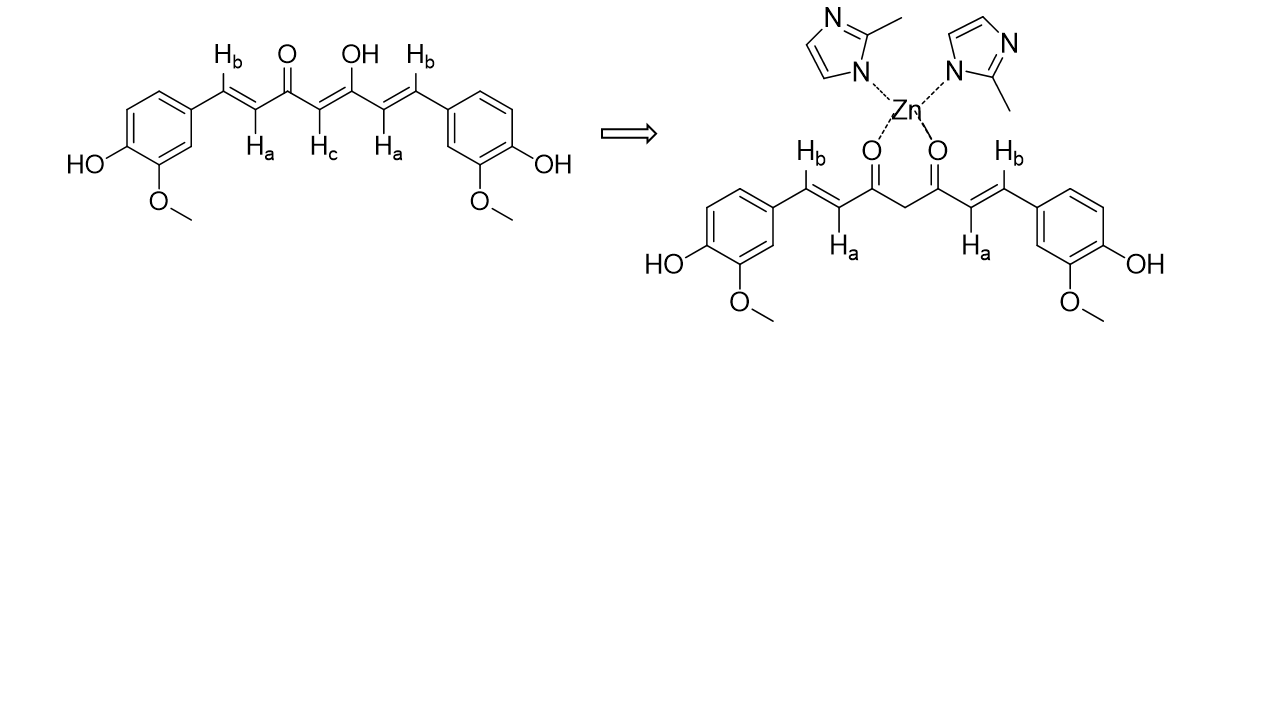


**Scheme 1:** Schematic representation of curcumin before and after encapsulated with ZIF-8.

Table S2: Numerical values calculated in ^1^H NMR spectra of curcumin and CCM-ZIF-8 in CD_3_OD and CF_3_COOD solution.

| Peak | Curcumin (CCM)  (CD_3_OD and CF_3_COOD) | | CCM-ZIF-8  (CD_3_OD and CF_3_COOD) | |
| --- | --- | --- | --- | --- |
|  | Δ Valve | J value | Δ Valve | J value |
| H_a_ | 6.60 | 15.8 | 6.60 | 15.8 |
| H_b_ | 7.55 | 15.8 | 7.56 | 15.8 |
| H_c_ | 5.93 | - | - | - |


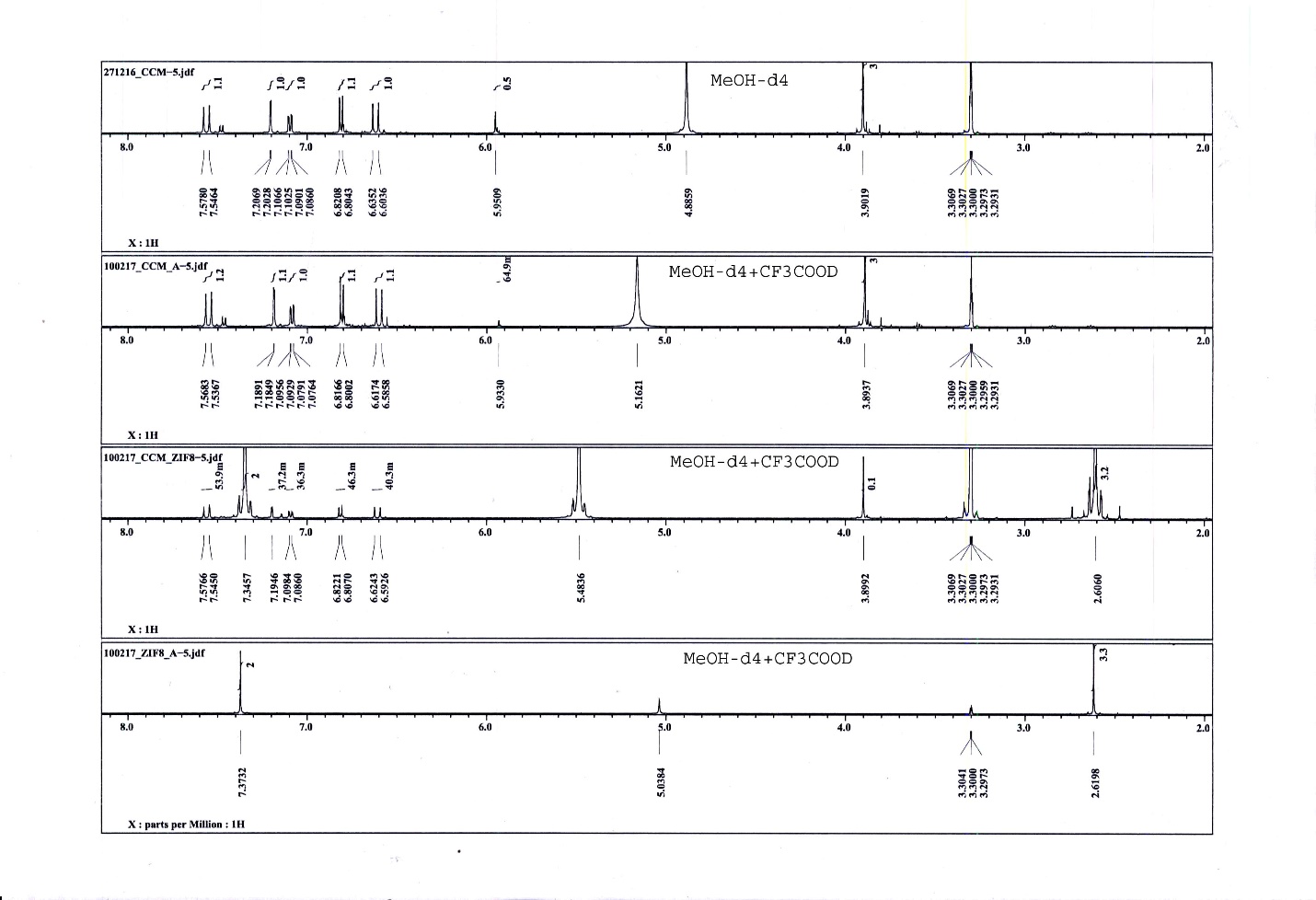


Figure S4: ^1^H NMR spectra of CCM (in pure CD_3_OD), CCM (in CD_3_OD and CF_3_COOD), ZIF-8 (in CD_3_OD and CF_3_COOD) and CCM-ZIF-8 (in CD_3_OD and CF_3_COOD).


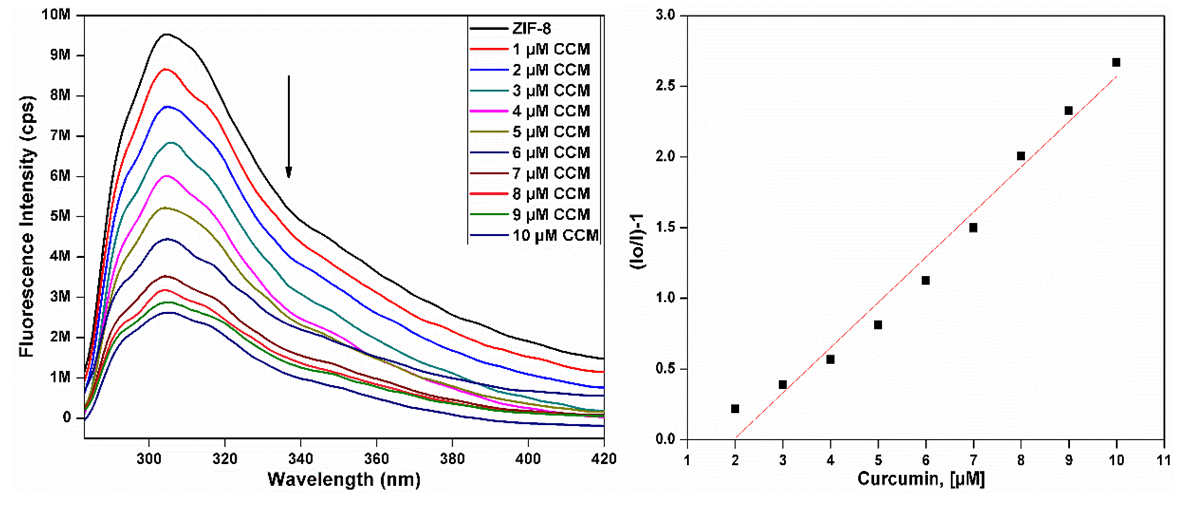


Figure S5: Fluorescence spectra of ZIF-8 at variousconcentrations of curcumin ranging from 1-10 µM. Fluorescence intensity ratio before (I_0_) and after (I) the addition of curcumin versus the concentration of curcumin. The concentration of ZIF-8 was 10 mg mL^-1^. The excitation wavelength was 250 nm. The regression coefficient and regression equationis R^2^=0.9745and y=0.3196+0.626x.


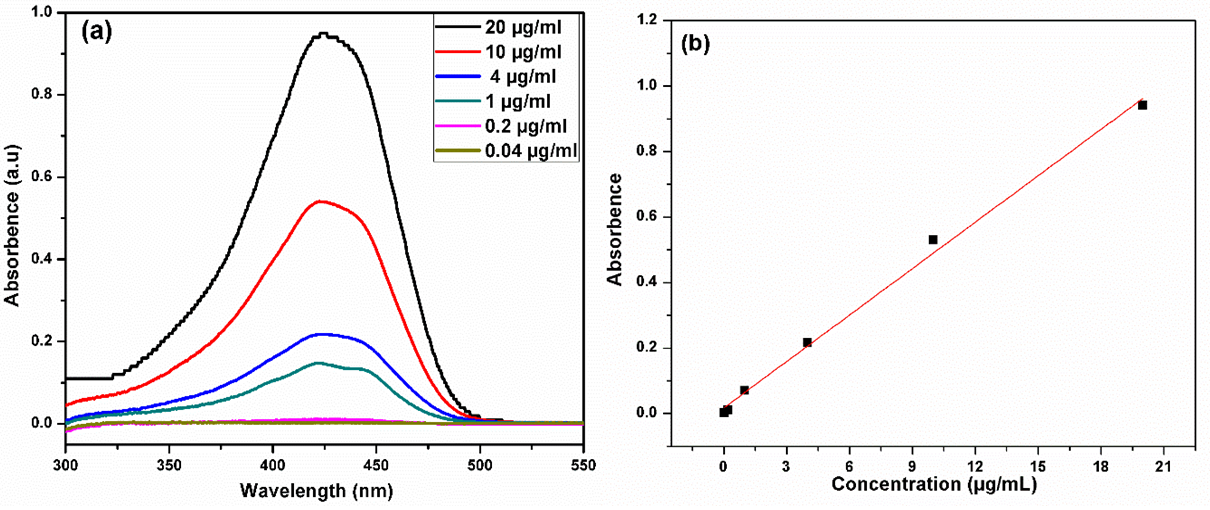


Figure S6: UV-Vis absorption spectra of curcumin in ethanol at different concentrations. The Linear relationship for concentration and absorbance intensities in ethanol, linear range of concentration is from 0.04-20µg/ml (R^2^=0.99482) and regression equation is y=0.01769+0.04724x.


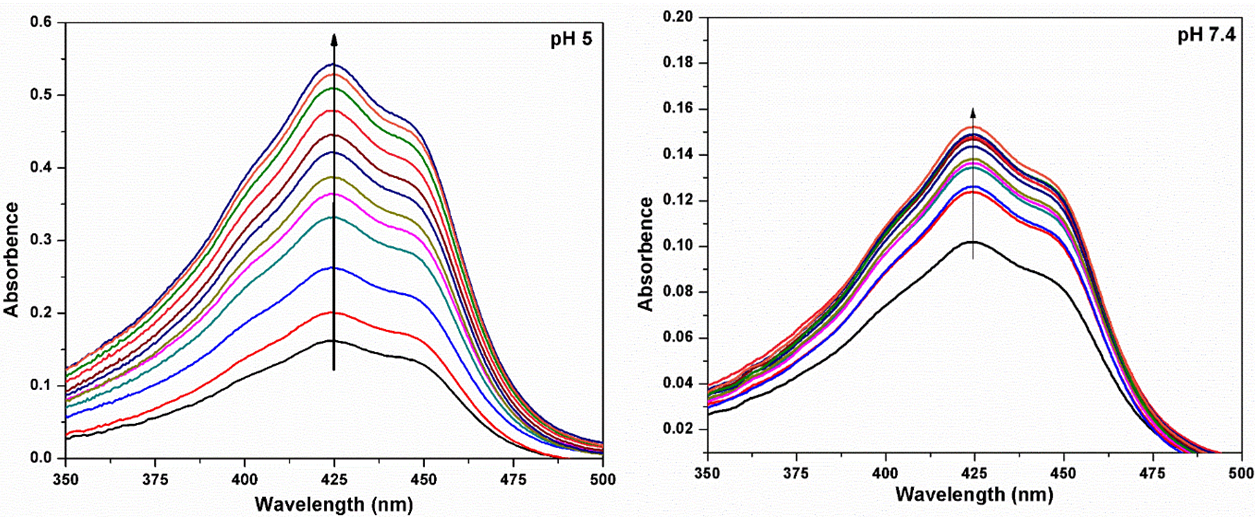


Figure S7: The absorption peaks of curcumin recorded at 425 nm in PBS solutions (pH 5 and 7.4) at selected time intervals from UV-Vis spectra during drug release studies.


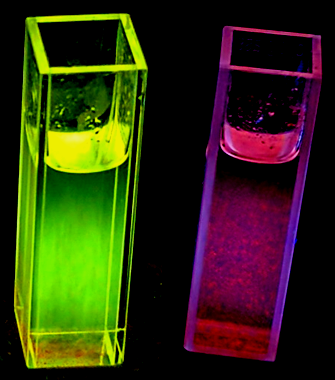


Figure S8: The optical images of curcumin and CCM-ZIF-8 under UV light excitation at 380 nm.


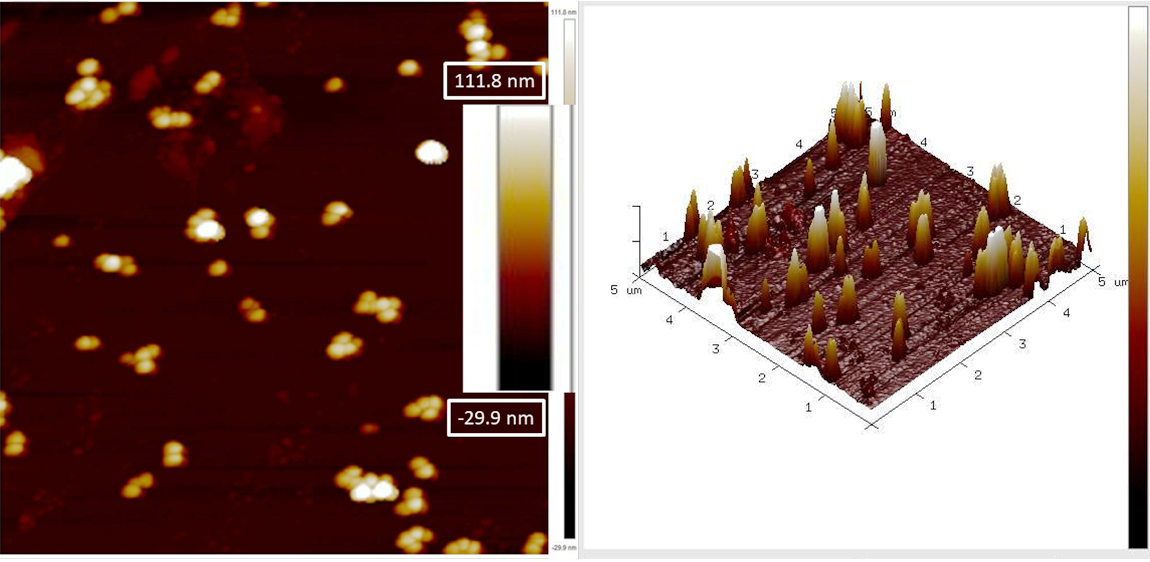


Figure S9:AFM images of CCM-ZIF-8.

**Reference**

1. Zhuang et al. "Optimized metal–organic-framework nanospheres for drug delivery: evaluation of small-molecule encapsulation." ACS nano 8.3 (2014): 2812-2819.
